# Localized primary amyloidosis of the breast: a case report and review of the literature

**DOI:** 10.1186/s12893-016-0178-6

**Published:** 2016-09-13

**Authors:** Wakako Tsuji, Eiji Takeuchi, Satoshi Oka, Taro Yamashita, Fumiaki Yotsumoto

**Affiliations:** 1 Department of Breast Surgery, Shiga Medical Center for Adults, Shiga, Japan; 2 Department of Pathology, Moriguchi Keijinkai Hospital, Osaka, Japan; 3 Department of Hematology and Oncology, Shiga Medical Center for Adults, Shiga, Japan; 4 Diagnosis Unit for Amyloidosis, Department of Neurology, Kumamoto University Hospital, Kumamoto, Japan

**Keywords:** Primary amyloidosis of the breast, Congo red, ALk, Amyloid, Breast cancer

## Abstract

**Background:**

Primary amyloidosis of the breast is an unusual benign disease that mostly occurs in postmenopausal elderly women. Amyloidosis is the deposition of amorphous protein within tissues. Breast biopsy is necessary to make a definite diagnosis in order to avoid unnecessary surgical methods. Localized primary amyloidosis of the breast has a good prognosis. However, secondary amyloidosis is a systemic disease and has a poor prognosis.

**Case presentation:**

We report the case of a 77-year-old female with primary amyloidosis of the breast. She noticed a lump in her left breast. Mammographic and ultrasonographic examinations indicated breast cancer. However, core needle biopsy showed amyloidosis, not cancer of the breast. For further examinations, the patient visited the outpatient clinics of the hematology, dermatology, and gastroenterology departments. She underwent bone marrow aspiration, computed tomography, cardiac ultrasonography, random skin biopsy, gastrofiberscopy, and colonofiberscopy. Plasma cell myeloma and systemic amyloidosis were ruled out, and localized breast amyloidosis was highly suspected. Lumpectomy was performed to make a definite diagnosis, and histological evaluations revealed that this patient had localized amyloidosis of the breast, and the deposited amyloid protein was of the amyloid light chain kappa type.

**Conclusions:**

Breast biopsy is necessary in order to avoid unnecessary surgical technique. A diagnosis should be achieved only through a histological evaluation. The main treatment of localized primary amyloidosis of the breast is surgical removal.

## Background

Amyloidosis of the breast is a very rare disease. We experienced a case of primary amyloidosis of the breast for the first time in 27 years at our institution. Amyloidosis of the breast was first reported in 1973 [1], and most of the reported cases involved postmenopausal elderly women. Breast amyloidosis can be a part of a systemic disease or may be localized to the breast. Furthermore, amyloidosis of the breast is reported to be often associated with an underlying breast cancer [2].

Primary amyloidosis of the breast typically presents with a painless and palpable mass [3]. The common mammographic findings of breast amyloidosis are multiple nodules with or without calcifications [2, 4–6]. O’Brien et al. reported that amyloidosis of the breast demonstrates low signal intensity on T1-weighed magnetic resonance imaging (MRI) and high signal intensity on T2-weighted MRI [7]. Reports on ultrasonographic findings of breast amyloidosis are rare [8].

Breast amyloidosis typically appears as diffuse breast involvement in the systemic form of amyloidosis, however, this condition can also be localized to the breast only. A study from the Mayo Clinic reviewed 40 cases of amyloidosis of the breast, and demonstrated that systemic amyloidosis accounted for nearly 50 % of all cases [3]. Moreover, concurrent hematologic malignancies were found in association with amyloidosis of the breast in 55 % of all patients.

Amyloidosis is classified as (i) primary amyloidosis and amyloidosis related to multiple myeloma (light chain amyloidosis [AL]), (ii) secondary amyloidosis (amyloid A amyloidosis [AA]), or (iii) familial amyloidosis (amyloidogenic transthyretin [ATTR]) on the basis of the chemical composition of the tumors. The most common type of amyloid is AL. The kappa light chain type is more frequently found than the lambda light chain type. Secondary amyloidosis is characterized by the deposition of the acute-phase protein serum amyloid A (SAA). Secondary amyloidosis is caused by an underlying chronic inflammatory disease such as rheumatoid arthritis or a malignant tumor. AA amyloid disappears after permanganic acid treatment. Familial amyloid polyneuropathy is one type of hereditary generalized amyloidosis. This disease is caused by a mutation in the *transthyretin* (*TTR*) gene, with the ATTR type being usually identified.

Breast biopsy is necessary to make a definite diagnosis of amyloidosis of the breast, in order to avoid unnecessary surgical interventions. Localized primary amyloidosis of the breast has a good prognosis. However, secondary amyloidosis is caused by a systemic disease and has a poor prognosis [9]. The main treatment of primary amyloidosis of the breast is surgical removal [4].

## Case presentation

A 77-year-old Japanese woman noticed a hard, non-tender lump in the left lower inner part of her breast and visited the outpatient clinic for breast surgery on September 24 2014. She had no family history of amyloidosis or breast cancer. She had hypertension, angina pectoris, and arteriosclerosis. She also had an operation history: abdominal total hysterectomy and bilateral salpingo-oophorectomy for myoma uteri at the age of 52 years.

**Fig. 1:**
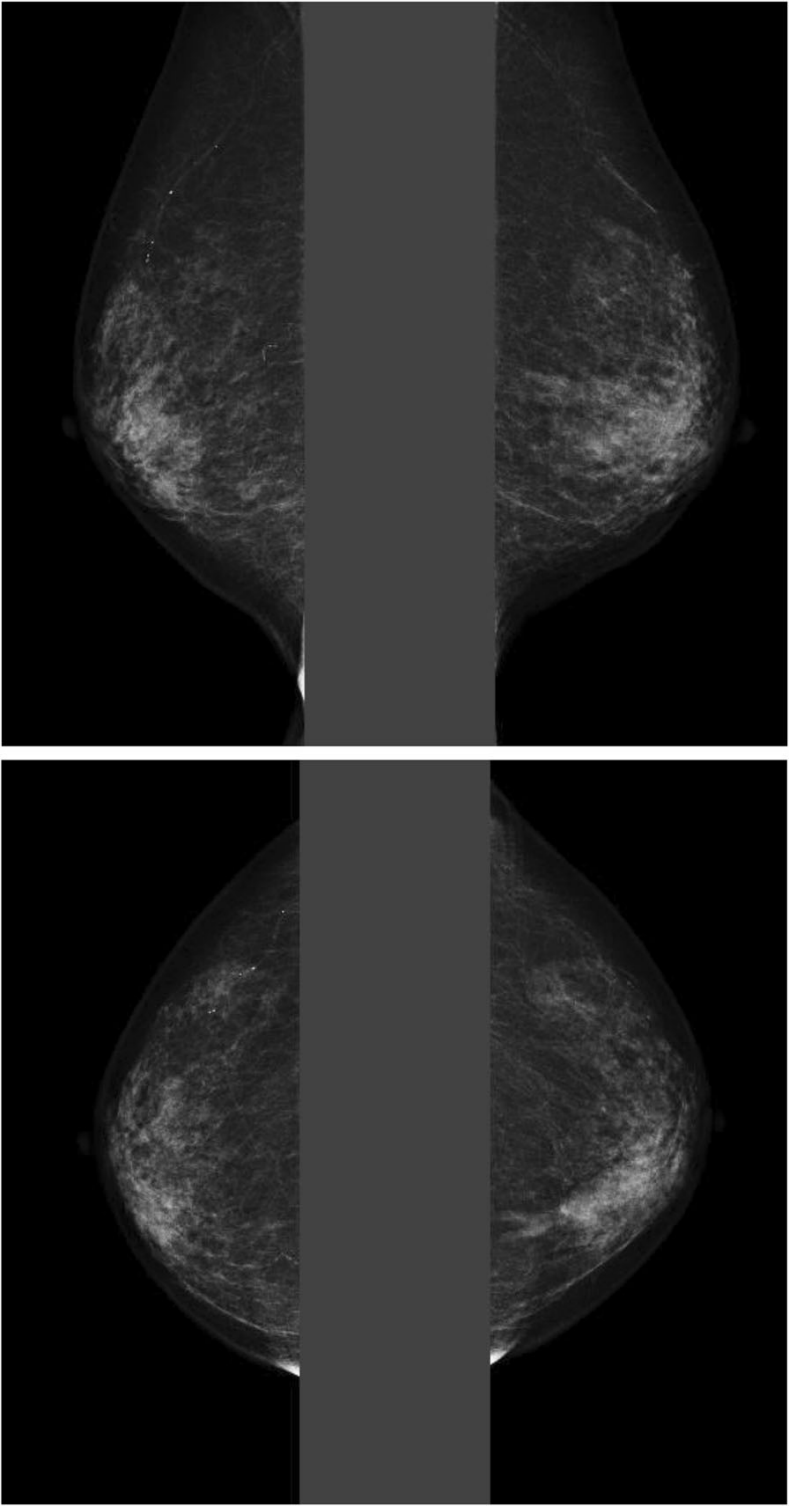
Mammogram findings. The bilateral craniocaudal view of the mammograms demonstrated focal asymmetry in the outer left breast. No pleomorphic calcifications was seen

**Fig. 2:**
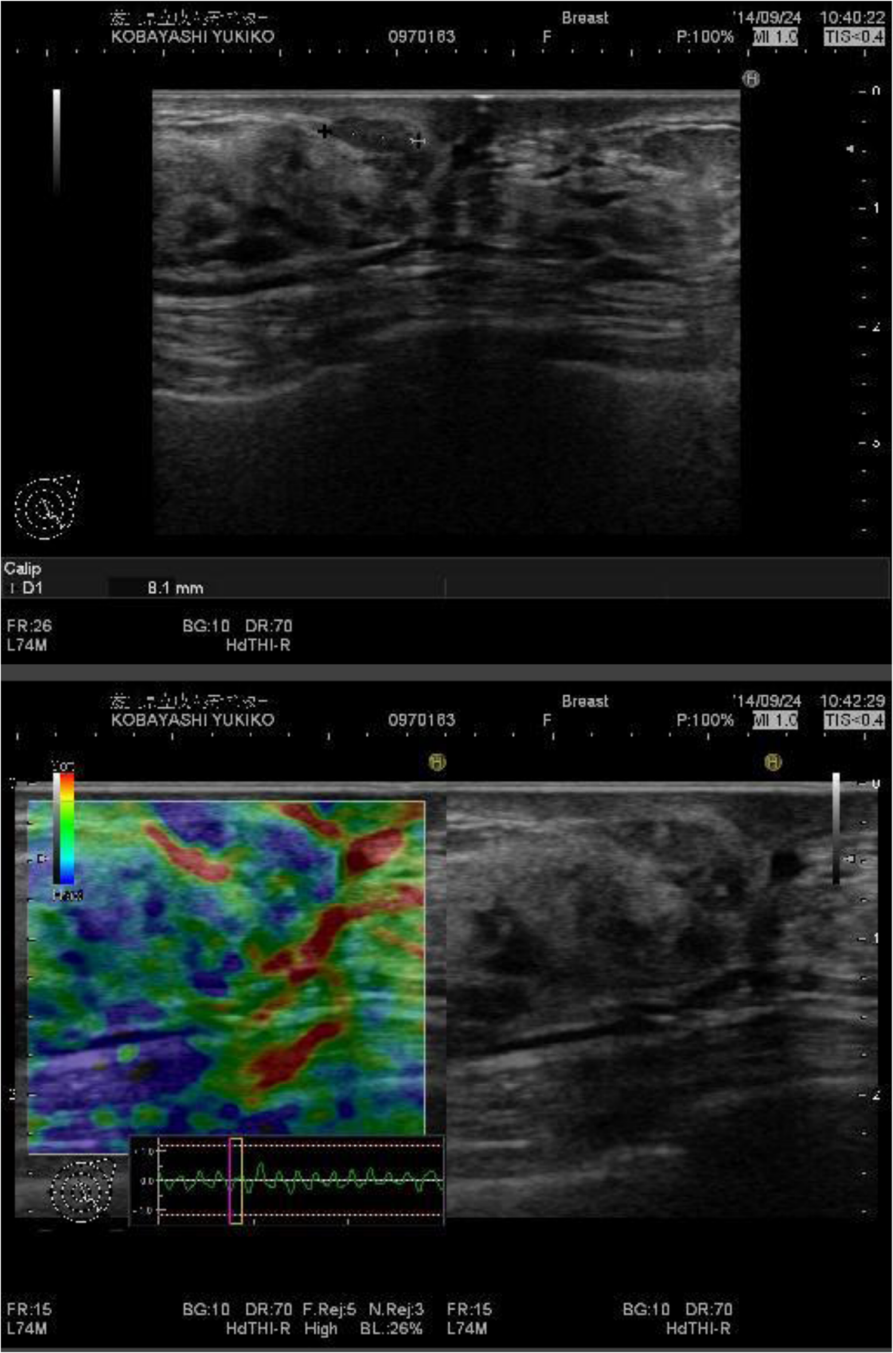
Ultrasonographic findings. Breast ultrasonogram showed an irregular low echoic area in the inner lower quadrant. No blood flows were present in the low echoic area, and elastography revealed that the area was soft unlike breast cancer

A hard lump was palpable on the left lower inner part of her breast. The mammogram showed no mass or pleomorphic calcifications in both breasts: however, a focal asymmetric density was observed on the inner side of the left breast in the craniocaudal view, which was assessed as BI-RADS category 3 (Fig. 1). On breast ultrasonogoraphy, an irregular low echoic area was observed at the site of the lump (Fig. 2). No blood flows were seen entering the low echoic area. Elastography (elasticity imaging technique) revealed that the low echoic area was soft, a finding that is different from that of breast cancer.

An ultrasound-guided core needle biopsy was done to rule out breast cancer. Hematoxylin and eosin staining revealed eosinophilic material in the extracellular area. The specimen was positive for Congo red staining even after permanganic acid treatment, and exhibited yellow-green birefringence under polarizing microscopy.

Further examinations were carried out to rule out plasma cell myeloma, other B cell tumors, or systemic amyloidosis according to National Comprehensive Cancer Network Clinical Practice Guidelines in Oncology. The patient had neither M-protein in serum immune electrophoresis nor Bence-Jones protein in urine immune electrophoresis examination. Bone marrow aspiration was negative for plasma cell myeloma or other B cell tumors. No lymphadenopathy was seen on computed tomography. Electrocardiogram and cardiac ultrasonography showed no evidence of cardiomyopathy or amyloidosis related changes. Stomach, rectum, bone marrow, and random skin biopsies were performed and none of biopsies showed evidence of amyloidosis. Taken together, systemic amyloidosis and multiple myeloma were ruled out, and localized breast amyloidosis was highly suspected.

**Fig. 3:**
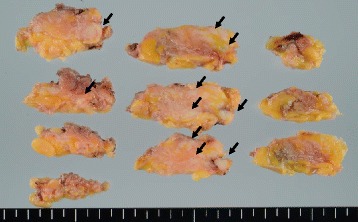
Macroscopic view of the specimen. The macroscopic breast specimens showed abnormal white masses as arrows indicate

**Fig. 4:**
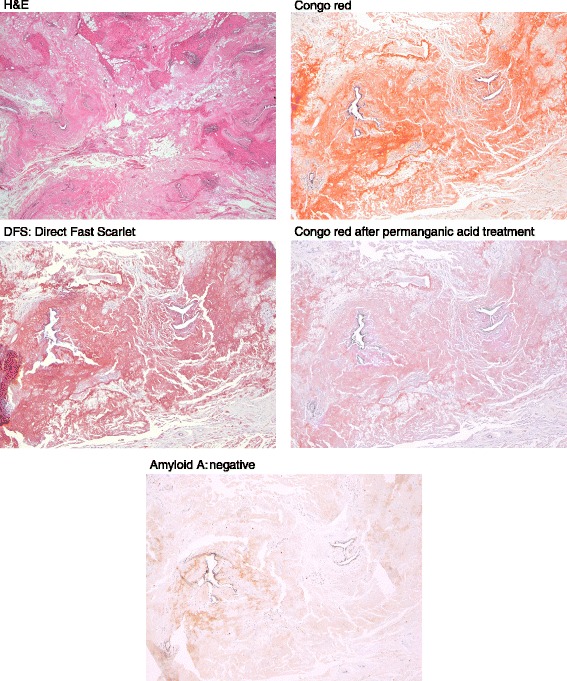
Microscopic observations (hematoxylin and eosin [H&E], direct fast scarlet [DFS], Congo red, Congo red after permanganic acid treatment, and amyloid L). H&E staining showed homogenous eosinophilic depositions with surrounding margins of breast parenchyma. No malignancy such as breast carcinoma, lymphoma, or plasma cell myeloma was seen. The deposits were positive to Congo red and DFS staining after permanganic acid treatment. Immunohistochemistry for AA amyloid was negative

To make a definite diagnosis, lumpectomy was performed on December 25, 2014. Macroscopic specimens of the mammary gland showed abnormal white masses as arrows indicate (Fig. 3). The final pathology demonstrated homogenous eosinophilic depositions with surrounding margins of breast parenchyma (Fig. 4). The deposits were positive to Congo red and direct fast scarlet (DFS) staining after permanganic acid treatment (Fig. 4). Immunohistochemistry for AA amyloid was negative. No malignancy such as breast carcinoma, lymphoma, or plasma cell myeloma was seen.

**Fig. 5:**
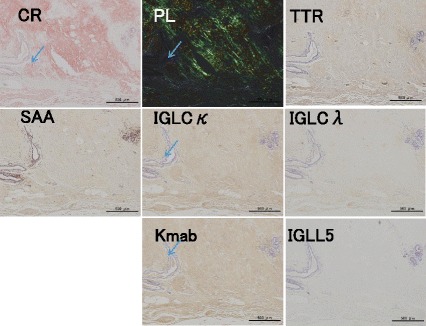
Immunohistochemistry. The resected breast tissue was positive for Congo red (CR), IgLCk (immunoglobulin light chain kappa [polyclonal]), and Kmab (immunoglobulin light chain kappa [monoclonal])(Fig. 5). Under polarized light, the protein exhibited yellow-green birefringence. The specimen was negative for SAA (serum amyloid A), TTR (transthyretin), IgLCλ (immunoglobulin light chain lambda [polyclonal]), and IGLL5 (immunoglobulin lambda-like polypeptide 5)

For further immunohistochemical staining, specimens were sent to the Department of Neurology of Kumamoto University. Amyloid typing with immunohistochemistry was performed using antibodies directed against the following antigens: polyclonal rabbit anti-human prealbumin (TTR) (DAKO), monoclonal mouse anti-human amyloid A (clone mc1) (DAKO), polyclonal rabbit anti-human kappa light chains (DAKO), polyclonal rabbit anti-human lambda light chains (DAKO), anti-human kappa light chain monoclonal antibody, clone H16-E (Novus Biologicals), anti-IGLL5 immunoglobulin lambda-like polypeptide 5, rabbit-Poly (Novus Biologicals), rabbit monoclonal antibody [EP1368Y] to apolipoprotein A-I (Abcam), and polyclonal rabbit anti-human beta-2-microglobulin (DAKO). The resected breast tissue was positive for Congo red, IgLCk (immunoglobulin light chain kappa [polyclonal]), and Kmab (immunoglobulin light chain kappa [monoclonal]) (Fig. 5). Under polarized light, the protein exhibited yellow-green birefringence. The specimen was negative for serum amyloid A (SAA), TTR, IgLCλ (immunoglobulin light chain lambda [polyclonal]), and IGLL5 (immunoglobulin lambda-like polypeptide 5) (Fig. 5). The amyloidosis was shown to be the ALk type, which is more frequently encountered than the ALλ type [10]. The patient was free from any lymphoid malignancies or immunological disorders. Therefore, the final diagnosis was primary amyloidosis of the breast.

The patient is currently being followed-up at our institution, and has shown no evidence of disease recurrence or breast cancer at 1 year after the surgery.

This report highlights imaging and histopathological assessment of amyloidosis of the breast. Amyloidosis of the breast is rare and should be distinguished from breast cancer, and histological assessment is necessary.

## Conclusions

Localized primary amyloidosis of the breast is a very rare benign disease characterized by abnormal protein deposition in the mammary glands. This case of primary breast amyloidosis was experienced for the first time in the recent 27 years at our institution. The current standard of care of primary breast amyloidosis is surgical resection, and systemic therapy is not necessary [4]. Localized primary amyloidosis of the breast has a good prognosis; however, the patient should be followed after surgery when recurrence or malignancy occurs.

## Abbreviations

AA: Amyloid type A
AL: Amyloid light chain
Ig: Immunoglobulin
TTR: Transthyretin.

## References

[1] Fernandez BB, Hernandez FJ (1973). Amyloid tumor of the breast. Arch Pathol.

[2] Charlot M, Seldin DC, O’Hara C, Skinner M, Sanchorawala V (2011). Localized amyloidosis of the breast: a case series. Amyloid.

[3] Said SM, Reynolds C, Jimenez RE, Chen B, Vrana JA, Theis JD, Dogan A, Shah SS (2013). Amyloidosis of the breast: predominantly AL type and over half have concurrent breast hematologic disorders. Mod Pathol.

[4] Huerter ME, Hammadeh R, Zhou Q, Weisberg A, Riker AI (2014). Primary amyloidosis of the breast presenting as a solitary nodule: case report and review of the literature. Ochsner J.

[5] Ngendahayo P, Faverly D, Herin M (2013). Primary breast amyloidosis presenting solely as nonpalpable microcalcifications: a case report with review of the literature. Int J Surg Pathol.

[6] Shim Y, Kim MJ, Ryu HS, Park SH (2013). Primary breast amyloidosis presenting as microcalcifications only. Korean J Radiol.

[7] O’Brien J, Aherne S, McCormack O, Jeffers M, McInerney D (2013). MRI features of bilateral amyloidosis of breast. Breast J.

[8] Eghtedari M, Dogan BE, Gilcrease M, Roberts J, Cook ED, Yang WT (2015). Imaging and Pathologic Characteristics of Breast Amyloidosis. Breast J.

[9] Gluck BS, Cabrera J, Strauss B, Ricca R, Brancaccio W, Tamsen A (2000). Amyloid deposition of the breast. AJR Am J Roentgenol.

[10] Rocken C, Kronsbein H, Sletten K, Roessner A, Bassler R (2002). Amyloidosis of the breast. Virchows Arch.

